# *Delonix regia* Leaf Extract (DRLE): A Potential Therapeutic Agent for Cardioprotection

**DOI:** 10.1371/journal.pone.0167768

**Published:** 2016-12-09

**Authors:** Lung-Shuo Wang, Chun-Ting Lee, Wei-Lieh Su, Shih-Che Huang, Shu-Chi Wang

**Affiliations:** 1 Department of Chinese Medicine, Tainan Sin-Lau Hospital, Tainan, Taiwan; 2 Department of Chinese Medicine, Kaohsiung Chang Gung Memorial Hospital, Kaohsiung, Taiwan; 3 Department of Occupational Therapy, Kaohsiung Medical University, Kaohsiung, Taiwan; 4 School of Medicine for International Students, I-Shou University, Kaohsiung, Taiwan; 5 Department of Internal Medicine, E-DA Hospital, Kaohsiung, Taiwan; 6 The School of Chinese Medicine for Post-Baccalaureate, I-Shou University, Kaohsiung, Taiwan; University of PECS Medical School, HUNGARY

## Abstract

*Delonix regia* (Boj. Ex. Hook) is a flowering plant in the pea family found in tropical areas and its leaves are used informally to treat diseases in folk medicine. However, the cardioprotective effects in this plant are still unclear. In this study, we found that the *Delonix regia* leaf extract (DRLE) (400 mg/kg/d) can reduce the mortality rate in an isoproterenol (ISO)-induced heart injury and hypertrophy mouse model. Decreased serum levels of creatine phosphokinase, LDH, GOT, TNF-alpha and increased nitric oxide levels were found in DRLE-treated ISO-injured mice. In the *in vitro* study, the porcine coronary artery exhibited vasodilation effect induced by DRLE in a dose-dependent manner. In the DRLE toxic test, overdose of DRLE showed the high safety in normal mice and may have the ability to remove the metabolic wastes in blood. In conclusion, we demonstrated for the first time that DRLE has the cardioprotective effects by activating the vasodilation through NO pathway and preventing the myocyte injury via inhibition of TNF-alpha pathway. We suggest that DRLE may act as a promising novel herbal medicine for cardioprotection.

## Introduction

*Delonix regia* (Bojer ex. Hook.) Raf., also known as the Royal Poinciana or Flamboyant, is a species of flowering plants in the pea family, Fabaceae and subfamily Caesalponioideae. It’s widely planted as ornamental trees in tropical areas, such as Taiwan, India, Vietnam, Malaysia, and the central region of South America. In some countries, *D*. *regia* has folkloric used as a medicinal agent to treat some disorders, such as constipation, inflammation, rheumatoid arthritis, diabetes, pneumonia, and malaria [1–6]. Many biological activity substances in the extracts of *D*. *regia* were reported to have anti-inflammatory [5, 6], antioxidant [4, 7, 8], antimicrobial [4, 9], anti-diarrhoeal [6, 10], antidiabetic [3], hepatopretective [11, 12], wound healing [13], and gastroprotective activity [14]. These functional phytoconstituents exist in leaves, flowers, barks, and seeds of *D*. *regia* including flavonoids, alkaloids, saponins, sterols, β-sitosterol, lupeol, tannins, carotenoids, and phenolic acids. [4, 6, 8, 15–17].

Ischemic heart disease (IHD) is a severe cardiovascular disease with a high global mortality rate. Previous studies have indicated that inflammation and oxidation of heart cells are commonly seen in IHD and contributed to IHD formation [18]. Several traditional Chinese prescriptions such as cinnamon, paeonol-danshensu combination, and catalpol from *Rehumannia glutinosa* were reported to be beneficial for acute myocardial injury [19–21]. In this study, we followed the law of Chinese medicine judging theory “*red foods nourish the heart*” on plant morphology, speculated that the flaming red flowers of *D*. *regia* may act as an “*activate blood and resolve stasis*” reagent on heart diseases and may improve the heart functions. Leaf extracts of *D*. *regia* (DRLE) was used as a tool for its convenient to obtain and the higher anti-inflammatory and antioxidative properties.

This study is the first time we demonstrate that the DRLE has the vasodilation effect on heart vessels and can act as a cardioprotective agent to protect the heart from isoproterenol-induced damage. Our findings may provide a possible candidate drug or healthy food for clinical medical use to treat or prevent cardiovascular diseases in the future.

## Materials and Methods

### Plant collection and preparation

The leaves of *D*. *regia* were collected from Kaohsiung, Taiwan and were taxonomically identified by botanist professor of Institute of Natural Products, College of Pharmacy, Kaohsiung Medical University, Kaohsiung, Taiwan. The samples were washed twice using double-distilled H_2_O, dried in shade, and ground into fine powder for further processing.

### Extraction of *D*. *regia* leaf

The *D*. *regia* leaf powder was mixed with 80% ethanol at a ratio of 1:5 (w/w) for 48 h, filtrated and collected the supernatant. The insoluble residue was then mixed with ethanol at a ratio of 1:3 (w/w) for 48 h and repeated again. The three collected supernatants were poured, ran for vacuum concentration and freeze-dried to give the product (yield 13.8 ± 2.4%; mean ± SD), then stored in dark bottles for further use.

### Animals

The (12- to 16-wk-old) C57BL/6 female mice were obtained from National Laboratory Animal Center, Taipei, Taiwan. The mice received regular mouse chow and tap water *ad libitum*. All the experimental protocols were reviewed and approved by the Committee of Institutional Animal Care and Use of I-Shou University (Approval No: IACUC-ISU-103005) and all the methods in this study were carried out in accordance with the approved guidelines. We followed the Guiding Principles for the Care and Use of Vertebrate Animals in Research and Training of the American Physiological Society during mice maintenance and experimentation.

### *In vivo* heart injury induction and DRLE treatment

The isoproterenol (ISO)-induced mice heart injury and hypertrophy model was performed in our experiment [22, 23] The leaf extract of *D*. *regia* (DRLE) was dissolved in ddH_2_O and administrated to mice orally in low and high doses (100 and 400 mg/kg/d, respectively) for consequent 9 days. The control group was given with water and no DRLE treatment. ISO was injected subcutaneously at a dose of 60 mg/kg/d at the Day 4 for consecutive 5 days. The mice serum at the beginning (Day 0) and the end before sacrificed (Day 9) were collected from the tail vein for advanced biochemical assay. Creatine phosphokinase (CPK), lactate dehydrogenase (LDH), glutamic oxaloacetic transaminase (GOT), and glutamic pyruvic transaminase (GPT) were evaluated by using a biochemical analyzer (ARKRAY SPOTCHEM EZ SP-4430). TNF-alpha and IL-10 were assayed by conducting ELISA (R&D Systems, Minneapolis, MN, USA). Nitric oxide concentration was measured using the Griess reaction [24]. The survival rate was recorded daily. At the end of the experiment, organs of heart, liver, and kidney were removed, fixed with 10% formaldehyde, embedded by paraffin and sliced with H&E staining for subsequent pathological examination.

### Oral toxicity assay

Mice in this test were fed 2 g/kg/d of DRLE orally for 14 days. Serum samples were collected from the tail veins at the beginning and the end of the experiment for biochemical assay. The CPK, LDH, GOT, GPT, blood urea nitrogen (BUN), total protein, creatinine, and uric acid were evaluated using a biochemical analyzer (ARKRAY SPOTCHEM EZ SP-4430) and compared with the reference values [25]. TNF-alpha and IL-10 were measured by conducting ELISA (R&D Systems) and the NO concentration was tested using the Griess reaction method [24]. The mice were sacrificed at Day 15 and tissues were obtained for pathological study in H&E staining.

### Isolation of porcine coronary artery rings

Explanted pig hearts, weighing approximately 110 kg, were obtained from a local abattoir. The heart was placed in an ice-cold Krebs–Henseleit buffer composed of the following (in mM): 122 NaCl, 4.7 KCl, 15.5 NaHCO_3_, 1.2 KH_2_PO_4_, 1.2 MgCl_2_, 1.8 CaCl_2_, and 11.5 glucose, with pH 7.4, and preserved on ice during transport to the laboratory approximately 60 min elapsed between the removal of the hearts and the arrival of the tissue in the laboratory. All procedures were performed in compliance with relevant laws and national guidelines.

The left anterior descending coronary artery (LAD) was excised, removed excess fat and connective tissue in an ice-cold buffer solution. Four 4-mm rings were obtained from each LAD, and were prepared for isometric contractile force recordings as described previously, but with minor modifications [26–30]. To control for the possible indirect effects of endothelium-derived vasoactive factors, the endothelium was removed physically by rubbing the luminal surface gently with a cotton swab. In preliminary experiments, removal of the endothelium was confirmed by the loss of relaxation in response to substance P (10 nM) [30].

### *In vitro* relaxation of isolated porcine coronary arterial rings caused by DRLE

The porcine coronary arterial rings were mounted using a wireless triangular support (Radnoti, Monrovia, CA, USA) in conjunction with a rigid L-shaped support. The triangular support was attached to a force-displacement transducer (FORT 10g, World Precision Instruments Inc., Sarasota, FL, USA) connected to an amplifier and a computer recording system (BIOPAC systems, Santa Barbara, CA, USA). Next, the coronary arterial rings were suspended in an organ bath containing 5 mL Krebs–Henseleit buffer and gassed with 95% O_2_•5% CO_2_. The final pH at 37°C was 7.40 ± 0.05.

The coronary arterial ring preparations were equilibrated at a resting tension of 2.0 g. After initial equilibration, the preparations were exposed to 60 mM KCl for 4 min. The experiments commenced after a 45-min equilibration period. For the relaxation measurements in the contracted arterial rings, the DRLE was added in a non-cumulative manner to U46619 (100 nM)-contracted muscle strips 15 min after the addition of U46619. The relaxation responses were represented as a percentage of U46619-stimulated contraction. Only one single dose response was studied with each preparation.

### Statistical analysis

Statistical analysis was conducted using GraphPad Prism 4 (San Diego, CA, USA). All data were expressed as mean ± standard deviation (SD). The differences between groups were performed using unpaired student’s t-test and one-way analysis of variance (ANOVA) with Tukey’s post hoc test. Significant difference was defined as *P* < 0.05, *P* < 0.01 or *P* < 0.001.

## Results

### DRLE enhances the survival rate in ISO-induced mice

**Fig 1:**
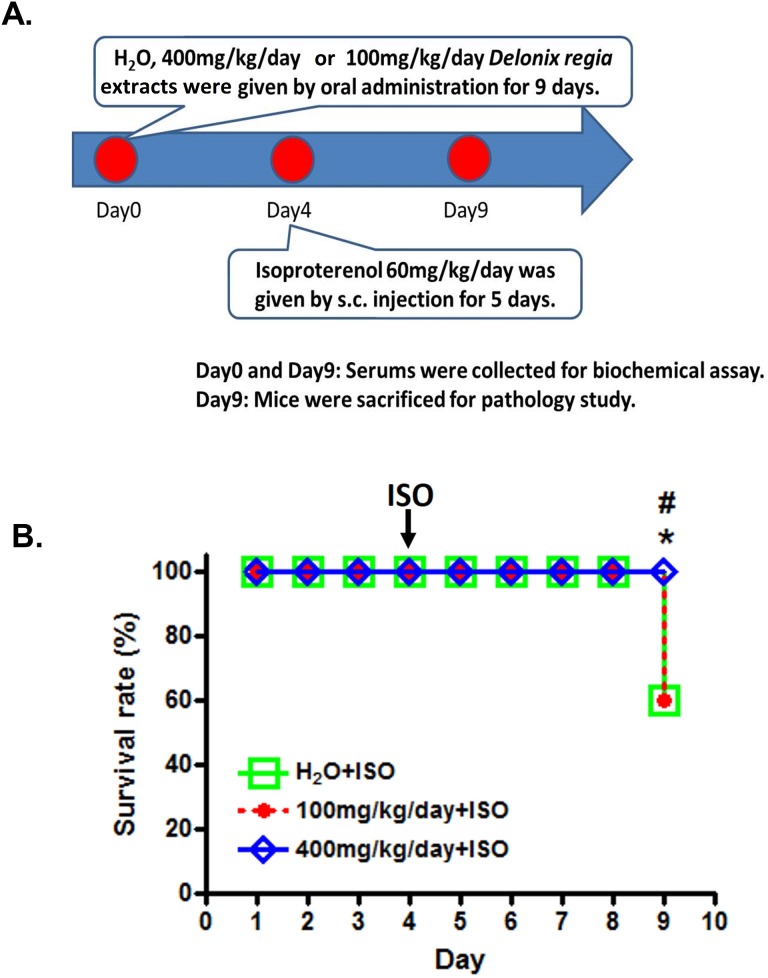
DRLE had a cardioprotective effect. (A) H_2_O and DRLE (100 mg/kg/d) and (400 mg/kg/d) were orally administered to C57BL/6 mice for 9 d. At Day 4, 60 mg/kg/d of isoproterenol was injected subcutaneously for 5 d. (B) The survival rate of ISO-injected mice treated with H_2_O and 100 mg/kg/d and 400 mg/kg/d of DRLE. *, *P* < 0.05 vs. H_2_O. ^#^, *P* < 0.05 vs. 100 mg/kg/d (n = 5 per group).

In this study, we first explored the survival rate of ISO-induced mice on DRLE treatment. The flowchart of the experimental design is shown in Fig 1A. The low, high doses of DRLE (100 and 400 mg/kg/d) and water were oral gavaged individually in ISO-treated mice for consecutive 9 days. At the 9th day, all ISO-induced mice treated with high dose of DRLE were alive (survival rate 100%), while the mice treated with low dose of DRLE and water displayed only 60% survival rates (Fig 1B). The results revealed that high dose (400 mg/kg/d) of DRLE can effective prevent ISO-induced mice from dying and enhance the survival chances.

### DRLE reduces ISO-induced ventricular hypertrophy and heart injury

**Fig 2:**
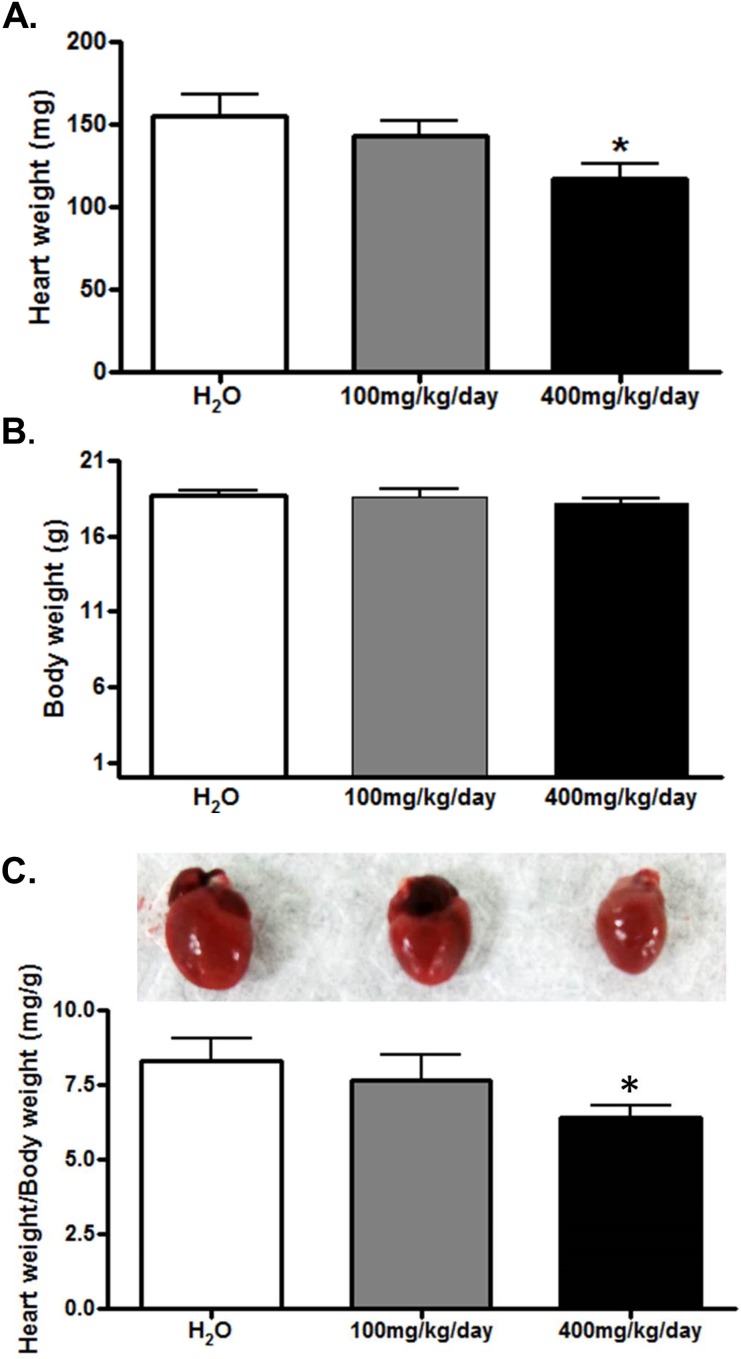
DRLE reduced ISO-induced ventricular hypertrophy. Heart and body weight in ISO-induced mice treated with H_2_O, 100 and 400 mg/kg/d DRLE (A) Heart weight. (B) Body weight. (C) The image of different sized mouse hearts and heart weight/body weight (mg/g) used to estimate the degree of ventricular hypertrophy. *, *P* < 0.05 vs. H_2_O (n = 5 per group).

**Fig 3:**
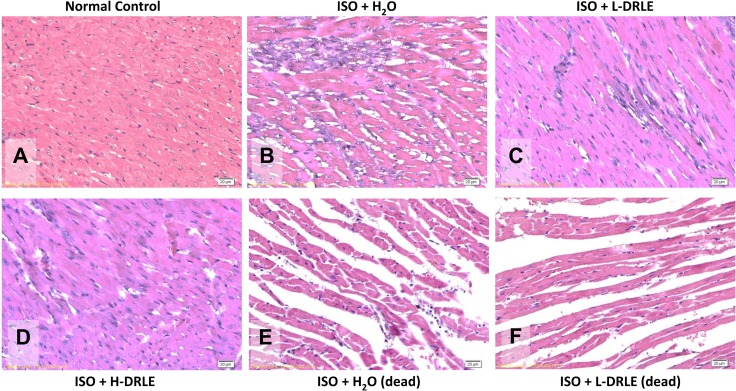
DRLE alleviated ISO-induced heart injury. Pathological examination of heart tissues in normal and ISO-induced mice fed with or without DRLE (H&E, 400X). (A) Normal control; (B) 60 mg/kg/d of ISO; (C) 60 mg/kg/d of ISO + 100 mg/kg/d of DRLE (L-DRLE); (D) 60 mg/kg/d of ISO + 400 mg/kg/d DRLE (H-DRLE); (E) 60 mg/kg/d of ISO (mice dead before sacrifice); and (F) 60 mg/kg/d of ISO + 100 mg/kg/d of DRLE (mice dead before sacrifice).

The heart weight and histopathological examination were performed to assess the protective effect of DRLE on ISO-induced heart injury [31, 32]. The heart-body weight ratio in mice treated with high dose DRLE (400 mg/kg) had the lowest proportion compared with the low dose (100 mg/kg) and water control groups (6.43 ± 0.82 vs. 7.65 ± 0.86 and 8.29 ± 1.50 kg, respectively, p< 0.05), and it displayed in a dose-dependent manner (Fig 2). In histological examination, normal cardiac myocyte was a single-nucleus, striated, and branched structure (Fig 3A). The injured myocytes by isoproterenol existed irregular and split arrangement, severe inflammation, infiltration of leukocytes, and lost the nucleus in the pathological morphology, which showed the sign of myocardial infarction (Fig 3B and 3E). The cell morphology in low dose DRLE-treated group was similar to the normal control and its inflammatory state was alleviated compared with non-treated heart injury group (Fig 3C and 3F). Surprisingly, the best protection was occurred in high dose DRLE-treated group (Fig 3D). It displayed effective protection against ISO-induced myocyte damage and inflammation, thus increased the survival rate. All these examinations revealed that the high dose DRLE can reduce ISO-induced ventricular hypertrophy and Injury, thus can restore heart to reach almost normal state.

### DRLE prevented ISO-induced elevation of CPK, LDH, and GOT

**Fig 4:**
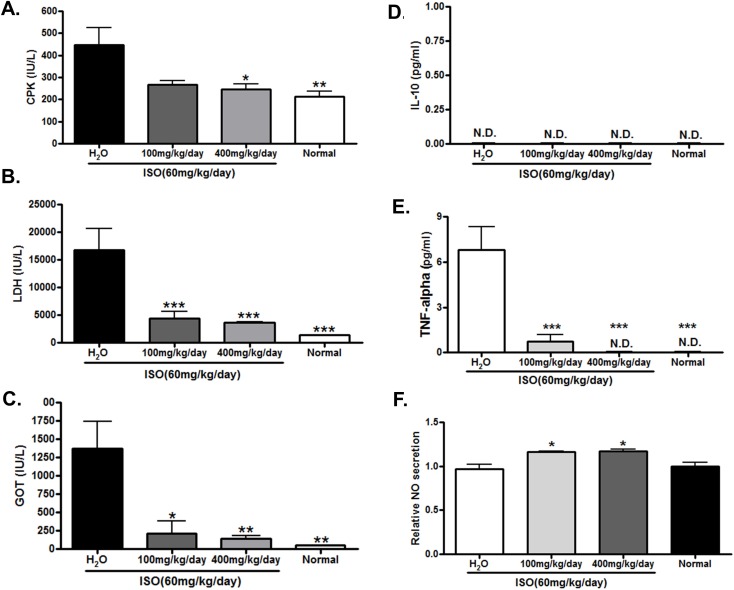
DRLE prevented ISO-induced elevation of CPK, LDH, GOT, and TNF-α, and elevated serum NO level. Serum levels of biomarkers and cytokines in normal and ISO-induced mice treated with H_2_O, 100 and 400 mg/kg/d DRLE. (A) CPK (B) LDH (C) GOT. (D) IL-10 (E) TNF-α (F) NO.*, *P* < 0.05 vs. H_2_O; **, *P* < 0.01 vs. H_2_O; ***, *P* < 0.001 vs. H_2_O (n = 3 to 5 per group).

To confirm our pathological finding, several heart injury markers were evaluated in different groups. In the ISO-induced heart injury group, serum CPK, LDH, and GOT were substantial elevated (0.45 ± 0.11, 16.7 ± 6.88, and 1.37 ± 0.63 IU/mL, respectively), which denote the inflamed and damaged sign. In the DRLE-treated mice, elevated serum CPK, LDH, and GOT levels were significantly decreased and appeared in a dose-dependent manner (Fig 4A, 4B and 4C). These data revealed that DRLE can restrain the elevation of CPK, LDH, and GOT by ISO, and can effective prevent the heart injury in IHD.

### DRLE reduced serum TNF-alpha and increased NO levels in ISO-induced mice *in vivo*

Previous researches showed that the inflammatory and cardiovascular change play critical roles in progressive heart injury and myocyte hypertrophy [21, 32–40]. In this study, we determined serum levels of IL-10, TNF-alpha, and NO to check whether the immunomodulation and vasodilation were involved in the protective effects of DRLE. As shown in Fig 4, serum TNF-alpha was decreased and serum NO was elevated in DRLE-treated mice, while the serum IL-10 was unable to detect because the very low levels in our study (Fig 4D, 4E and 4F). These evidences confirmed that the cardioprotective effect of DRLE may be accomplished through immunomodulation and vasodilation routes via TNF-alpha and NO secretion pathways.

### DRLE had a vasodilator effect *in vitro*

**Fig 5:**
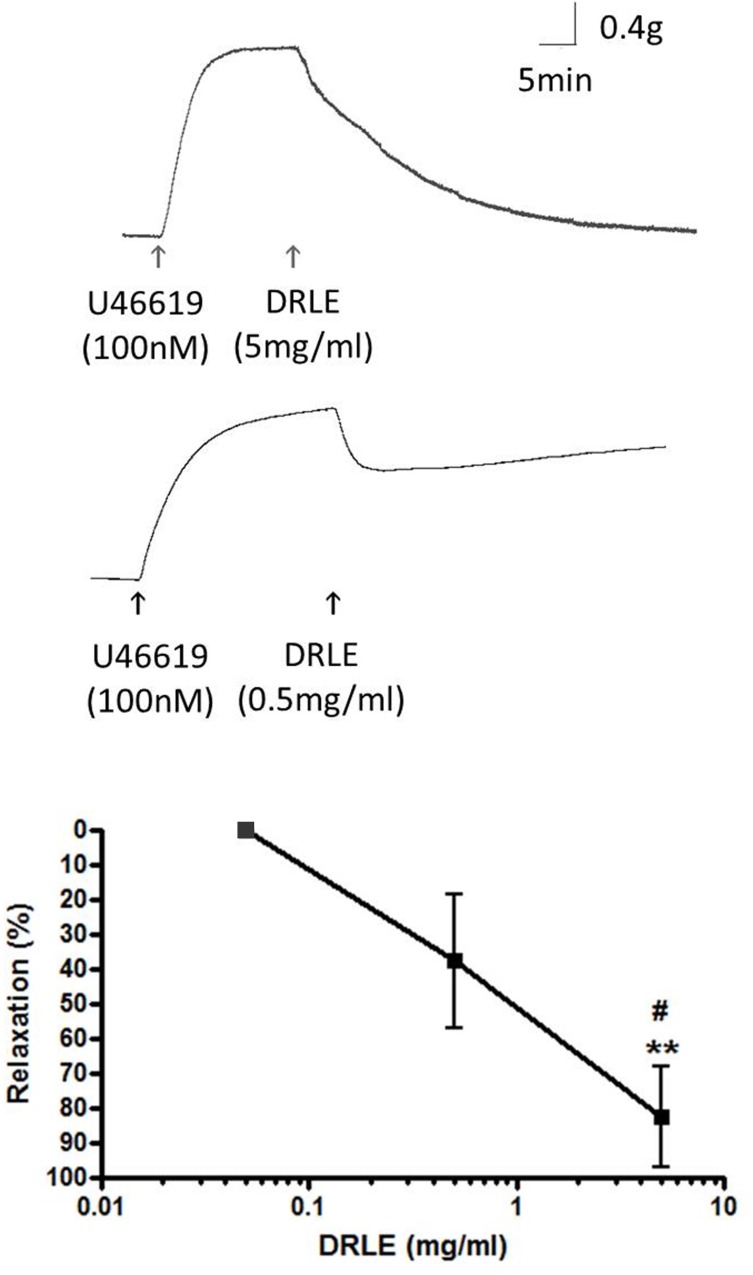
DRLE had a direct vasodilatory effect on porcine coronary arterial rings. Porcine left arterial descending coronary arteries were treated with different DRLE doses of 0.05 mg/mL, 0.5 mg/mL, and 5 mg/mL to observe the direct vasodilatory effect. **, *P* < 0.01 vs. 0.05 mg/mL; #, *P* < 0.05 vs. 0.5 mg/mL (n = 3 to 5 per group).

To determine the vessel relaxation potency of DRLE *in vitro*, we used porcine LAD as study material. Maximum vasoconstriction was first reached by U46619, an analog of endoperoxide prostaglandin PGH_2_, then added different doses of DRLE (5, 0.5 and 0.05 mg/mL, from high to low) to test the relaxation degree. The vessels displayed the largest relaxation in 5 mg/mL DRLE treatment (82.5 ± 28.7%), followed by 0.5 mg/mL DRLE (37.6 ± 27.2%), while the lowest dose of DRLE had no any vasodilator effect (0.0 ± 0.0%, in 0.05 mg/mL) (Fig 5). It showed a dose-dependent manner. The results verified that the DRLE had a direct effect on vasodilation *in vitro* and this was corresponding to the cardioprotective effect *in vivo*.

### DRLE exhibited a high safety profile

Shewale, et al. had reported that the DRLE was safe and non-toxic in the acute toxicity studies *in vivo* [5]. However, the chronic toxicity of DRLE remained unclear. In this study, a five times high dose of DRLE (2 g/kg) was administrated orally for consecutive 14 days and test the toxicity in mice. In the whole process, no lethality or any toxic reaction was found, and mice displayed normal in hair colour, activity and appearance. In addition, after overdose DRLE treatment, serum CPK, LDH, GOT, BUN, and uric acid levels were decreased while total protein level was increased compared with untreated control group. No change was detected in body weight, heart weight, heart-body weight ratio, GPT, albumin, creatinine, IL-10, TNF-alpha, and relative NO secretion levels (Table 1). All these biochemical data were in the normal range and not influenced by overdose DRLE, proved that DRLE is safe and harmless not only in acute state but also in chronic consumption.

**Table 1:** Body Weight and Serum Biochemical Markers in Oral Toxicity Test of DRLE.

	Normal	DRLE (2g/kg/d)	Reference values
Body Weight (g)	20.8 ± 1.3	20.8 ± 1.3	19.3–24.4
Heart Weight (mg)	124 ± 12	130 ± 18	–
Heart Weight / Body Weight (mg/g)	5.95 ± 0.36	6.41 ± 0.77	4–7
CPK (IU/L)	212 ± 71	150 ± 51*	105–649
LDH (IU/L)	1334 ± 342	672 ± 94.6*	293–3150
GOT (IU/L)	48.1 ± 16.5	25.0 ± 3.54*	10–122
GPT (IU/L)	14.9 ± 0.5	15.2 ± 1.0	10–73
Total protein (g/dl)	4.20 ± 0.84	5.60 ± 0.54*	3.5–8.3
Albumin (g/dl)	1.84 ± 0.65	1.90 ± 0.65	2.0–4.8
BUN (mg/dl)	32.0 ± 5.7	17.6 ± 2.5*	10–58.1
Creatinine (mg/dl)	0.52 ± 0.19	0.56 ± 0.11	0.3–1
Uric acid (mg/dl)	4.70 ± 0.76	3.44 ± 0.78*	2–12

Data are expressed as mean ± S.D.

* *P* < 0.05 vs. normal group

## Discussion

*Delonix regia* leaves are informal used to treat disorders in folk, but few studies have reported to elucidate their potential mechanisms [6]. In this study, we tested the DRLE functions on ISO-induced heart injury mice. The ISO-induced heart injury mice model is a reliable, reproducible, and commonly used method for human myocardial infarction studies [19–21, 36, 41]. In this model, both male and female have been used in studies for the gender factors seldom affect the heart functions in young animals. Female mice were adopted in our study instead of male to avoid the stress fighting in cage and cause unnecessary injury and death. The ISO-injured myocyte was suffer from the threats of inflammation, reduced nitric oxide secretion and increased free radicals generation, thus induce the heart injury and hypertrophy [20–22, 34, 37, 42].

In this study, leaves of *D*. *regia* were used instead of flowers, barks or seeds for its easier to obtain and exhibit the higher biological activities [4, 6]. Previous studies have identified that four major bioactivity phytoconstituents of DRLE were involved: (1) β-sitosterol, (2) lupeol, (3) flavonoids, and (4) phenolic acid (gallic acid, protocatehuic acid, and salicylic acid) [4–6, 12] (S4 Table, S1 Fig). (1) β-Sitosterol is a sterol of plants that can reduce cholesterol serum levels, regulate inflammation, and has anticancer activity [43–45]. β-sitosterol can also provide the antioxidative and cardioprotective effect by attenuating TNF-alpha-induced monocyte adhesion on human aortic endothelial cells [46, 47]. (2) Lupeol, a triterpenoid compound, has anti-inflammatory activity and may act as a promising compound to protect heart from injury [48–50]. (3) Flavonoids are potent compounds that exhibit antioxidant, anti-tumour, anti-inflammatory, and cardioprotective effects [51–58]. (4) Phenolic acids is abundant in DRLE and it exhibits the high antioxidant, anticancer, antimicrobial, and cardioprotective activity [4, 59–63]. Based on these studies, we surmise that the cardiovascular protection of DRLE may be the combination of the four bioactivity compounds, but which compounds arouse the major cardioprotective effect remain to be further investigation.

In the choice of DRLE dosage, we followed the previous *in vivo* studies and set a high (400 mg/kg/d) and low (100 mg/kg/d) doses of DRLE to perform our study [1, 3, 5, 12]. The results revealed that the high dose of DRLE can effective protect mice from death elicited by ISO-induced heart injury and hypertrophy, but low dose and ISO-induced control mice showed little or no function on it. The heart-body weight ratio and morphology of cardiac myocytes in high dose DRLE group revealed similar and regular arrangement to those of normal mice, but the myocytes in low dose DRLE and ISO-induced control mice still showed severe hypertrophy and injury.

For the reason that if excessive DRLE can cause toxic damage on normal mice, an overdose (2 g/kg/d) of DRLE was oral administrated consequently for 14 days to test the toxicity and safety in mice. In the whole process, mice showed healthy and vitality state. Serum biochemical data were all in the normal range. These results prove that DRLE is safe by oral route in mice. According to the safety test, we can make sure that the 400 mg/kg/d dose DRLE in our test can’t make any change in the normal mice. Another benefit in the DRLE toxic test is the reduced levels of serum CPK, LDH, GOT, BUN, and uric acids. It means the DRLE may has the potential to lower the metabolic wastes in serum and can act as a heathy food or reagent for clinical use.

In the results in biochemical assays, serum CPK, LDH, and GOT levels were significant reduced after high dose DRLE treatment. This is agreed with the normalized myocyte morphology and elevated survival rates in DRLE treatment. It means that the DRLE may benefit for the body operations. In this present study, low dose of DRLE has slightly improved but incompletely protected on cardiac functions. The high dose of DRLE exhibited the effective functions to protect heart from injury and thus elevated the high survival rate. In the histopathological results, cardiac myocytes in low dose DRLE group was still in a bad condition but appeared to be much better than the untreated ISO-induced heart injury group. The regular and perfect morphology was seen in high dose DRLE group and similar to the normal tissue. It means that high DRLE can protect the heart from worsen induced by ISO and restore to the almost normal state.

To elucidate the possible mechanism of DRLE on cardioprotection, serum makers such as TNF-alpha, IL-10, and NO were evaluated, which may play a critical role in heart injury and hypertrophy [64]. In our current results, we found that serum NO level was increased in DRLE-treated heart-injury mice. Previous study in eNOS-overexpressed mouse model had proved that released NO can attenuate ISO-induced hypertrophy [42]. Serum NO level raised by cinnamic acid and cinnamic aldehyde in other study had also indicated the cardioprotective effects on ISO-induced acute myocardial ischemia rats [21]. The eNOS upregulation can also be incited by Matrine in ISO-induced myocardial ischemia rats [37]. The eNOS activity was undetected in our study for the technique shortage, but the serum NO was significant elevated in ISO-injury mice after high dose DRLE treatment. However, there was no elevated serum NO secretion observed in normal mice treated with DRLE. We suggest that DRLE-induced NO secretion was triggered by ISO-induced heart injury. In the porcine LAD test *in vitro*, we found the obvious vasodilation effect after DRLE treatment. Thus we can confirm that the cardioprotective effect of DRLE in ISO-induced heart injury may partially triggered via NO secretion pathway.

**Fig 6:**
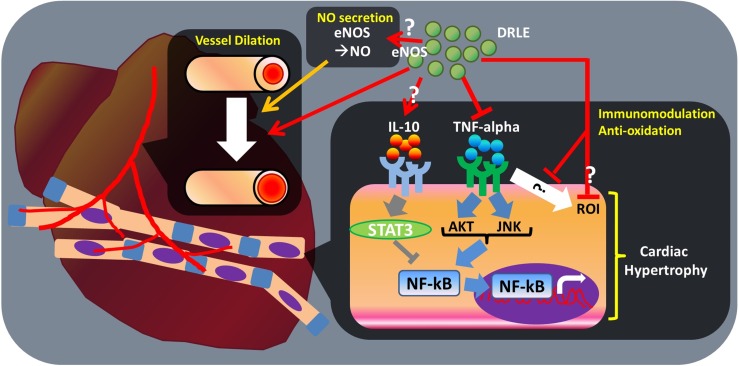
Possible cardioprotective mechanism of DRLE. Inflammation, oxidation, and NO secretion have been demonstrated to play a critical role in cardiac hypertrophy and myocardial injury. IL-10 can activate STAT3 to inhibit the NF-kB signalling pathway, thereby attenuating cardiac hypertrophy. TNF-alpha is associated with the AKT and JNK pathways in enhancing NF-kB activation to augment cardiac hypertrophy. TNF-alpha can also upregulate ROI expression to enhance myocyte hypertrophy. Another study reported that eNOS upregulation can attenuate myocardial injury. In our study, we first found that DRLE can attenuate TNF-alpha secretion, which might reduce AKT, JNK, and NF-kB signalling pathways, thereby inhibiting cardiac hypertrophy. ROI expression might also be inhibited by DRLE through TNF-alpha stimulation. We assumed that the direct inhibition of ROI is a potential mechanism for inhibiting cardiac hypertrophy, because the antioxidative effect of DRLE has also been reported. Second, we cannot confirm whether DRLE enhances IL-10 expression, because serum IL-10 level was undetected in our experiment. Third, the NO serum level increased in our study, but we could not determine the origin of the NO, and we postulated that eNOS is a possible candidate. Finally, we found that DRLE can dilate the porcine coronary artery directly *in vitro*.

Some inflammatory cytokines play a critical role in heart injury and hypertrophy, such as TNF-alpha and IL-10 [32, 35, 38–40]. TNF-alpha secretion was elevated in myocyte hypertrophy by generating reactive oxygen intermediates and activating AKT, JNK and NF-kappaB (NF-kB) pathways, thus caused myocyte damage [35, 38, 39]. Previous study indicated that TNF-alpha expression was reduced by cinnamic acid and cinnamic aldehyde in ISO-induced heart injury rats [21]. In our present data, serum TNF-alpha levels were significant decreased in ISO-induced heart injury mice after DRLE treatment. TNF-alpha had the downregulation effect on eNOS expression in heart failure patients and obese mice [33, 65]. Combined with the elevated serum NO level and reduced TNF-alpha levels in our results, we can affirm that the protection mechanism of DRLE may functioned via TNF-alpha and NO pathways. In addition, serum IL-10 overexpression can attenuate ventricular hypertrophy via the STAT3 pathway [32]. Although the serum IL-10 level was too low to be detected in our experiment, we can’t exclude the actions of IL-10 in heart tissues. The outline of the possible mechanisms of DRLE on cardioprotection is shown in Fig 6.

**Fig 7:**
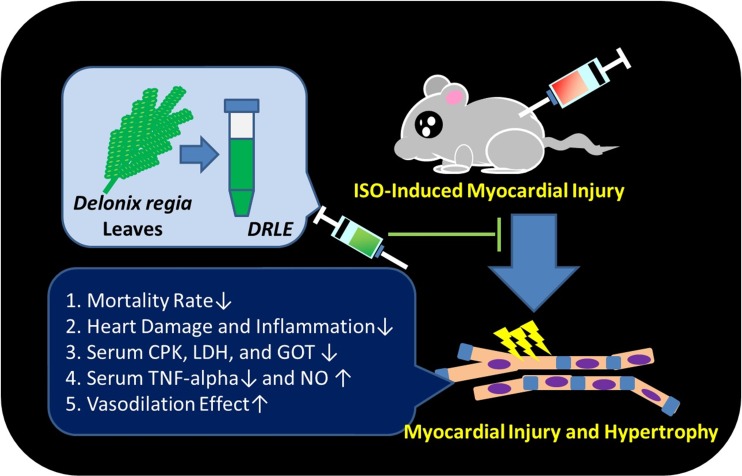
Cardioprotective effect of DRLE. In this study, we found that DRLE can reduce the mortality rate, cardiac hypertrophy and heart injury in ISO-injured mice. Serum CPK, LDH, GOT, and TNF-alpha were also reduced by DRLE administration. Serum NO levels were induced by ISO injection in mice *in vivo*, and had the vasodilatory effects *in vitro*.

In summary, we found that the DRLE can reduce the mortality rate and heart injury in ISO-induced IHD mice. The cardioprotective mechanisms of DRLE may act via the anti-inflammatory and vasodilation effects on myocytes which were mediated by TNF-alpha and NO secretions (Fig 7). We also found that DRLE was safe by oral route for at least two weeks and it may possess the potential to improve the heart, liver or kidney functions. These results proved that the DRLE can act as a promising cardioprotective candidate agent or healthy food to prevent or improve the heart damage in IHD. Further studies will be explored to find out the real bioactive compounds in DRLE which were involved in the improvement of heart functions.

## Supporting Information

S1 Table Body and heart weight in the ISO-induced mice with or without DRLE treatment. (DOCX)

S2 Table Biomarkers in ISO-induced mice with or without DRLE treatment. (DOCX)

S3 Table Serum cytokine and NO concentration levels induced by ISO with or without DRLE treatment. (DOCX)

S4 Table The four major compounds in DRLE. (DOCX)

S1 Fig The four major bioactivity compounds in DRLE. In previous studies, four major compounds in DRLE had been identified, including β-sitosterol, lupeol, flavonoids and phenolic acid. The background photograph of flowers was taken in Tainan, Taiwan, by Chun-Ting Lee, one of the co-authors in this article.
(TIF)
