# Supplementary material for: Delonix regia Leaf Extract (DRLE): A Potential Therapeutic Agent for Cardioprotection
Source: PLoS One. 2016 Dec 9;11(12):e0167768. doi: 10.1371/journal.pone.0167768 (PMC5147973; doi:10.1371/journal.pone.0167768)
Supplement: S1 Table — (DOCX) [file pone.0167768.s001.docx]

**S1 Table.** Body and heart weight in the ISO-induced mice with or without DRLE treatment.

|  | H_2_O + ISO | L-DRLE + ISO | H-DRLE + ISO |
| --- | --- | --- | --- |
| Body Weight (g) | 18.7 ± 0.7 | 18.6 ± 1.1 | 18.1 ± 0.8 |
| Heart Weight (mg) | 156 ± 25 | 143 ± 20 | 117 ± 19* |
| Heart Weight /Body Weight (mg/g) | 8.29 ± 1.50 | 7.65 ± 0.86 | 6.43 ± 0.82* |
| Data are expressed as mean ± S.D. * *P* < 0.05 vs. H_2_O group  L-DRLE: 100 mg/kg/d DRLE fed by oral gavage for consequent 9 days  H-DRLE: 400 mg/kg/d DRLE fed by oral gavage for consequent 9 days | | | |
