# Supplementary material for: Delonix regia Leaf Extract (DRLE): A Potential Therapeutic Agent for Cardioprotection
Source: PLoS One. 2016 Dec 9;11(12):e0167768. doi: 10.1371/journal.pone.0167768 (PMC5147973; doi:10.1371/journal.pone.0167768)
Supplement: S2 Table — (DOCX) [file pone.0167768.s002.docx]

**S2 Table.** Biomarkers in ISO-induced mice with or without DRLE treatment.

|  | H_2_O + ISO | L-DRLE + ISO | H-DRLE + ISO | Normal |
| --- | --- | --- | --- | --- |
| CPK (IU/L) | 448 ± 110 | 267 ± 32 | 246 ± 49* | 212 ± 71** |
| LDH (IU/L) | 16,715 ± 6,884 | 4,418 ± 2,169*** | 3,614 ± 229*** | 1,334 ± 342*** |
| GOT (IU/L) | 1,372 ± 635 | 212 ± 293* | 138 ± 81.4** | 48.1 ± 16.5** |
| Data are expressed as mean ± S.D. * *P* < 0.05 vs. H_2_O group; ** *P* < 0.01 vs. H_2_O group  L-DRLE: 100 mg/kg/d DRLE fed by oral gavage for consequent 9 days  H-DRLE: 400 mg/kg/d DRLE fed by oral gavage for consequent 9 days | | | | |
