# Supplementary material for: Delonix regia Leaf Extract (DRLE): A Potential Therapeutic Agent for Cardioprotection
Source: PLoS One. 2016 Dec 9;11(12):e0167768. doi: 10.1371/journal.pone.0167768 (PMC5147973; doi:10.1371/journal.pone.0167768)
Supplement: S3 Table — (DOCX) [file pone.0167768.s003.docx]

**S3 Table.** Serum cytokine and NO concentration levels induced by ISO with or without DRLE treatment.

|  | H_2_O + ISO | L-DRLE + ISO | H-DRLE + ISO | Normal |
| --- | --- | --- | --- | --- |
| IL-10 (pg/ml) | N.D. | N.D. | N.D. | N.D. |
| TNF-α (pg/ml) | 6.78 ± 3.08 | 0.74 ± 0.87*** | N.D.*** | N.D.*** |
| Relative NO secretion | 0.97 ± 0.08 | 1.17 ± 0.01* | 1.17 ± 0.03* | 1.00 ± 0.08 |
| Data are expressed as mean ± S.D. * *P* < 0.05 vs. H_2_O group; *** *P* < 0.001 vs. H_2_O group  L-DRLE: 100 mg/kg/d DRLE fed by oral gavage for consequent 9 days  H-DRLE: 400 mg/kg/d DRLE fed by oral gavage for consequent 9 days | | | | |
