# Supplementary material for: Delonix regia Leaf Extract (DRLE): A Potential Therapeutic Agent for Cardioprotection
Source: PLoS One. 2016 Dec 9;11(12):e0167768. doi: 10.1371/journal.pone.0167768 (PMC5147973; doi:10.1371/journal.pone.0167768)
Supplement: S4 Table — (DOCX) [file pone.0167768.s004.docx]

**S4 Table.** The four major compounds in DRLE.

| **Compounds** | **Cardioprotective effect** | **References*** |
| --- | --- | --- |
| β-sitosterol | 1. It is a sterol of plants that can reduce cholesterol serum levels, regulate inflammation, and has anticancer properties. 2. It could attenuate TNF-alpha-induced monocytal adhesion on human aortic endothelial cells. 3. It might involve antioxidative action. | [1-5] |
| Lupeol | It can reduce inflammation and is also a promising compound for protecting the heart from injury. | [6-8] |
| Flavonoids | Flavonoids are potent compounds that improve health, and exhibit anti-oxidant, anti-tumour, anti-inflammatory, and cardioprotective effects. | [10-16] |
| Phenolic acid | Studies have already indicated that phenolic acids can act as anti-oxidant, anti-cancer, anti-microbial, and cardioprotective agents. | [17-21] |

* References are listed as below.

References list:

1. Miettinen TA, Puska P, Gylling H, Vanhanen H, Vartiainen E: **Reduction of serum cholesterol with sitostanol-ester margarine in a mildly hypercholesterolemic population**. *The New England journal of medicine* 1995, **333**(20):1308-1312.
2. Bouic PJ, Lamprecht JH: **Plant sterols and sterolins: a review of their immune-modulating properties**. *Alternative medicine review : a journal of clinical therapeutic* 1999, **4**(3):170-177.
3. Bouic PJ: **Sterols and sterolins: new drugs for the immune system?** *Drug discovery today* 2002, **7**(14):775-778.
4. Loizou S, Lekakis I, Chrousos GP, Moutsatsou P: **Beta-sitosterol exhibits anti-inflammatory activity in human aortic endothelial cells**. *Molecular nutrition & food research* 2010, **54**(4):551-558.
5. Ganapathy P, Rajadurai M, Ashokumar N: **Effect of β-Sitosterol on Cardiac Troponins, Marker Enzymes and Biochemical Parameters in Isoproterenol-Induced Myocardial Infarction.** *Journal of Academia and Industrial Research* 2014, **3**(4):209-214.
6. Arul V, Kumaraguru S, Dhananjayan R: **Effects of ageline and lupeol, the two cardioactive principles isolated from the leaves of Aegle marmelos Corr.** *JOURNAL OF PHARMACY AND PHARMACOLOGY* 1999, **51**:252.
7. Geetha T, Varalakshmi P: **Anti-inflammatory activity of lupeol and lupeol linoleate in rats**. *Journal of ethnopharmacology* 2001, **76**(1):77-80.
8. Maity P, Hansda D, Bandyopadhyay U, Mishra DK: **Biological activities of crude extracts and chemical constituents of Bael, Aegle marmelos (L.) Corr**. *Indian journal of experimental biology* 2009, **47**(11):849-861.
9. Williams RJ, Spencer JP, Rice-Evans C: **Flavonoids: antioxidants or signalling molecules?** *Free radical biology & medicine* 2004, **36**(7):838-849.
10. Lotito SB, Frei B: **Consumption of flavonoid-rich foods and increased plasma antioxidant capacity in humans: cause, consequence, or epiphenomenon?** *Free radical biology & medicine* 2006, **41**(12):1727-1746.
11. Izzi V, Masuelli L, Tresoldi I, Sacchetti P, Modesti A, Galvano F, Bei R: **The effects of dietary flavonoids on the regulation of redox inflammatory networks**. *Front Biosci (Landmark Ed)* 2012, **17**:2396-2418.
12. Gomes A, Couto D, Alves A, Dias I, Freitas M, Porto G, Duarte JA, Fernandes E: **Trihydroxyflavones with antioxidant and anti-inflammatory efficacy**. *Biofactors* 2012, **38**(5):378-386.
13. Romagnolo DF, Selmin OI: **Flavonoids and cancer prevention: a review of the evidence**. *Journal of nutrition in gerontology and geriatrics* 2012, **31**(3):206-238.
14. Siasos G, Tousoulis D, Tsigkou V, Kokkou E, Oikonomou E, Vavuranakis M, Basdra EK, Papavassiliou AG, Stefanadis C: **Flavonoids in atherosclerosis: an overview of their mechanisms of action**. *Current medicinal chemistry* 2013, **20**(21):2641-2660.
15. Grassi D, Desideri G, Ferri C: **Flavonoids: antioxidants against atherosclerosis**. *Nutrients* 2010, **2**(8):889-902.
16. Fuhrman B, Aviram M: **Flavonoids protect LDL from oxidation and attenuate atherosclerosis**. *Current opinion in lipidology* 2001, **12**(1):41-48.
17. Barros L, Duenas M, Ferreira IC, Baptista P, Santos-Buelga C: **Phenolic acids determination by HPLC-DAD-ESI/MS in sixteen different Portuguese wild mushrooms species**. *Food and chemical toxicology : an international journal published for the British Industrial Biological Research Association* 2009, **47**(6):1076-1079.
18. Carocho M, Ferreira IC: **The role of phenolic compounds in the fight against cancer--a review**. *Anti-cancer agents in medicinal chemistry* 2013, **13**(8):1236-1258.
19. Alves MJ, Ferreira IC, Froufe HJ, Abreu RM, Martins A, Pintado M: **Antimicrobial activity of phenolic compounds identified in wild mushrooms, SAR analysis and docking studies**. *Journal of applied microbiology* 2013, **115**(2):346-357.
20. Heleno SA, Martins A, Queiroz MJ, Ferreira IC: **Bioactivity of phenolic acids: metabolites versus parent compounds: a review**. *Food chemistry* 2015, **173**:501-513.
21. Leifert WR, Abeywardena MY: **Cardioprotective actions of grape polyphenols**. *Nutr Res* 2008, **28**(11):729-737.
