# Supplementary figures and images for: Delonix regia Leaf Extract (DRLE): A Potential Therapeutic Agent for Cardioprotection
Source: PLoS One. 2016 Dec 9;11(12):e0167768. doi: 10.1371/journal.pone.0167768 (PMC5147973; doi:10.1371/journal.pone.0167768)

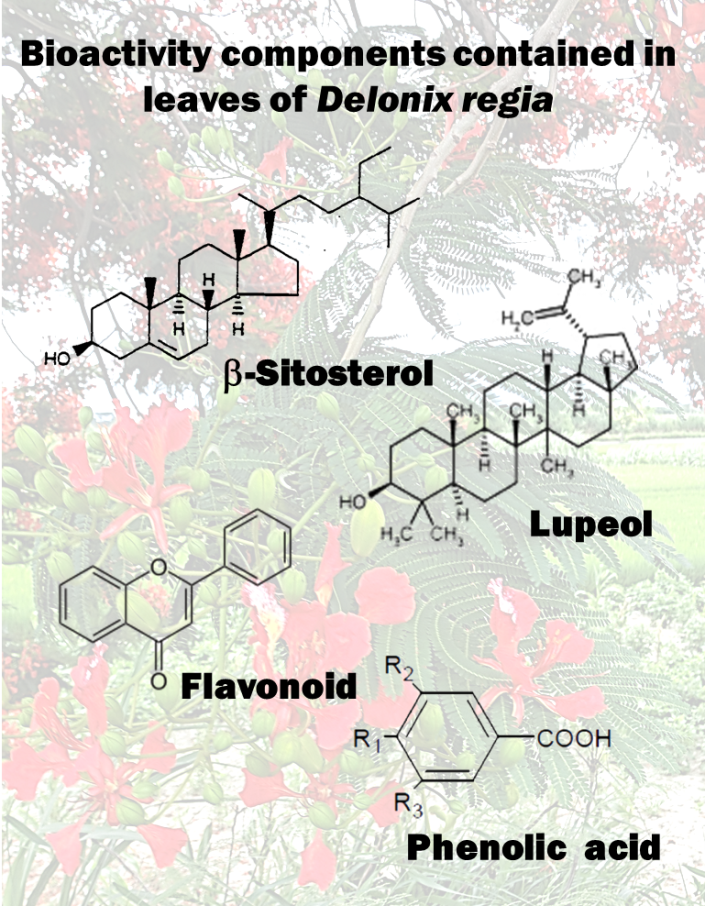

Supplement: S1 Fig — In previous studies, four major compounds in DRLE had been identified, including β-sitosterol, lupeol, flavonoids and phenolic acid. The background photograph of flowers was taken in Tainan, Taiwan, by Chun-Ting Lee, one of the co-authors in this article. (TIF) [file pone.0167768.s005.tif]
